# A general charge transport picture for organic semiconductors with nonlocal electron-phonon couplings

**DOI:** 10.1038/s41467-021-24520-y

**Published:** 2021-07-12

**Authors:** Weitang Li, Jiajun Ren, Zhigang Shuai

**Affiliations:** MOE Key Laboratory of Organic OptoElectronics and Molecular Engineering, Department of Chemistry, Tsinghua University, Beijing, China

**Keywords:** Electronic properties and materials, Computational methods

## Abstract

The nonlocal electron-phonon couplings in organic semiconductors responsible for the fluctuation of intermolecular transfer integrals has been the center of interest recently. Several irreconcilable scenarios coexist for the description of the nonlocal electron-phonon coupling, such as phonon-assisted transport, transient localization, and band-like transport. Through a nearly exact numerical study for the carrier mobility of the Holstein-Peierls model using the matrix product states approach, we locate the phonon-assisted transport, transient localization and band-like regimes as a function of the transfer integral (*V*) and the nonlocal electron-phonon couplings (Δ*V*), and their distinct transport behaviors are analyzed by carrier mobility, mean free path, optical conductivity and one-particle spectral function. We also identify an “intermediate regime” where none of the established pictures applies, and the generally perceived hopping regime is found to be at a very limited end in the proposed regime paradigm.

## Abstract (web summary)

Though research efforts have focused on elucidating the charge transport mechanism in crystalline organic semiconductors, a general transport model is still lacking. Here, the authors report a theoretical study of the carrier mobility in Holstein-Peierls model applied to organic semiconductors.

## Introduction

The last two decades have witnessed the rapid development of high-mobility crystalline organic semiconductors^1–3^. The first proposition of using Marcus’ semiclassical hopping model coupled with density functional theory to design high mobility molecules has been very successful and popular^4^. It can be regarded as the strong local electron–phonon coupling (EPC) limit, which was later improved by considering quantum nuclear effect and delocalization effect^5,6^, through which, isotope effect is found to be always negative and the dynamic disorder does not play appreciable role with some experimental supports^7–10^. However, such local EPC picture is challenged by the recently established transient localization (TL) model which invokes nonlocal EPC^11–14^. In due course, the molecular design principles derived from TL such as suppressing intermolecular vibration, which are quite different from the local picture, have proved successful in a number of experiments^15,16^. The applicability of the TL picture, nevertheless, is restricted to the regime of moderate transfer integral (electronic coupling) *V* and strong nonlocal EPC. Since EPC is a complicated many-body problem, it is highly desirable to present a general transport picture taking both local and nonlocal EPC into considerations in a rigorous way, instead of uncontrolled approximations.

Many recent efforts have been devoted to developing approximate methods that are able to portray a broader parameter space, including the band-like (BL) conduction regime^17,18^. Besides, unlike in the case of Holstein model in which it is beyond doubt that local EPC represents an obstacle for carrier diffusion^19–21^, how nonlocal EPC affects mobility does not have a definitive answer and the interplay between the local and nonlocal EPC is unclear. Early theoretical treatments for carrier mobility in crystalline organic semiconductors such as the Munn-Silbey approach and the polaron transformation often reach the conclusion that the nonlocal EPC leads to phonon-assisted (PA) transport and enhances mobility^22–24^, in sheer contrast with the basic starting point of the TL scenario. The findings of several numerical studies on the carrier mobility of organic semiconductors also contradict with the TL theory^8,25^. In principle, PA, TL, and BL are all possible mechanisms for charge transport with nonlocal EPC, valid at their respective parameter regimes, yet a universal theoretical treatment for the role of nonlocal EPC is not available due to the complex many-body electron–phonon interaction.

In this work, we present a nearly exact study of the charge transport mechanism in the Holstein–Peierls model using the time-dependent finite temperature matrix product state (MPS) formalism^26,27^. By studying EPC effect on the carrier mobility, mean free path, optical conductivity, and one-particle spectral function, we have located the PA, TL, and BL regimes simultaneously on the transfer integral – nonlocal EPC strength plane. We have also identified an intermediate regime where none of the existing pictures is truly applicable, as a generalization of the hopping-band crossover in the Holstein model.

## Results

### System Hamiltonian and Kubo Formula

We take the following one-dimensional Holstien–Peierls model with nearest-neighbor interaction and periodic boundary condition:

1 $$\hat{H}	=\; {\hat{H}}_{{\rm{e}}}+{\hat{H}}_{{\rm{ph}}}+{\hat{H}}_{{\rm{e-ph}}}\\ {\hat{H}}_{{\rm{e}}} 	=-V\mathop{\sum}\limits_{n}({c}_{n+1}^{\dagger }{c}_{n}+{c}_{n}^{\dagger }{c}_{n+1})\\ {\hat{H}}_{{\rm{ph}}}	= \mathop{\sum}\limits_{n,m}{\omega }_{m}{b}_{n,m}^{\dagger }{b}_{n,m}+\mathop{\sum}\limits_{n}{\omega }_{\theta }{b}_{n,\theta }^{\dagger }{b}_{n,\theta }\\ {\hat{H}}_{{\rm{e-ph}}}	= \mathop{\sum}\limits_{n,m}{g}_{m,{\rm{I}}}{\omega }_{m}({b}_{n,m}^{\dagger }+{b}_{n,m}){c}_{n}^{\dagger }{c}_{n}\\ 	+\mathop{\sum}\limits_{n}{g}_{\theta ,{\rm{II}}}{\omega }_{\theta }({b}_{n,\theta }^{\dagger }+{b}_{n,\theta })({c}_{n+1}^{\dagger }{c}_{n}+{c}_{n}^{\dagger }{c}_{n+1})$$

where *c*^†^ (*c*) and *b*^†^ (*b*) are the creation (annihilation) operator for electron and phonon respectively, and *V* is the intermolecular transfer integral. The electronic motion is limited to single-electron manifold. *ω*_*m*_ and *g*_*m*,I_ are the frequency and the dimensionless EPC constant of the *m*th intramolecular vibration mode. *ω*_*θ*_ and *g*_*θ*,II_ are the intermolecular vibration counterparts. *ℏ* is set to 1. The thermal fluctuation Δ*V* is related to intermolecular coupling constant *g*_*θ*,II_ by^28^:

2 $${{\Delta }}V={g}_{\theta ,{\rm{II}}}{\omega }_{\theta }\sqrt{\coth \frac{{\omega }_{\theta }}{2{k}_{B}T}}$$

The one-dimensional model in Eq. (1) is an approximation to realistic organic semiconductors, which typically adopt two-dimensional transport network^8,13,29^. In the Supplementary Fig. 4 we demonstrate that this approximation is valid for anisotropic materials by comparing the simulated one-particle spectral function with experimental angle resolved ultraviolet photoemission spectra (ARUPS)^30^, and at the end of the section we go beyond the one-dimensional model to discuss the isotropy effect on the different transport regimes.

In order to elucidate how nonlocal EPC affects charge transport at different transport regimes we focus on the role of transfer integral *V* and nonlocal EPC constant *g*_*θ*,II_ (or equivalently Δ*V* at a given *T*). Other parameters are fixed throughout this paper unless otherwise specified with values drawn from representative organic semiconductors. In organic semiconductors, the most common values of *V* and Δ*V* range from 10 meV to 150 meV and from 10 meV to 60 meV, respectively^31–33^. The intramolecular vibration frequency *ω*_*m*_ and local EPC constant *g*_*m*,I_ are taken from our previous DFT calculations for rubrene and the total 3*N* − 6 normal vibration modes are reduced to four effective modes^6,21^, namely *ω*_*m*_ = 26 meV, 124 meV, 167 meV, and 198 meV, with the corresponding dimensionless *g*_*m*,I_ 0.83, 0.26, 0.34, and 0.37, respectively. The intermolecular vibration frequency *ω*_*θ*_ is set to be 50 cm^−1^ (6.2 meV) as commonly adopted in literature^13,17,34^. The system is translational-invariant and we do not consider the effect of both diagonal and off-diagonal static disorder here^35^.

The carrier mobility is obtained via the Kubo formula^36^:

3 $$\mu =\frac{1}{{k}_{B}T{e}_{0}}\int_{0}^{\infty }\left\langle \hat{j}(t)\hat{j}(0)\right\rangle {\mathrm{d}}t=\frac{1}{{k}_{B}T{e}_{0}}\int_{0}^{\infty }C(t){\mathrm{d}}t$$

where for the Holstein–Peierls Hamiltonian in Eq. (1) the current operator $\hat{j}$ takes the form:

4 $$\hat{j}=\frac{{e}_{0}R}{i}\mathop{\sum}\limits_{n}\left[-V+{g}_{\theta ,{\rm{II}}}{\omega }_{\theta }({b}_{n,\theta }^{\dagger }+{b}_{n,\theta })\right]({c}_{n+1}^{\dagger }{c}_{n}-{c}_{n}^{\dagger }{c}_{n+1})$$

Here *R* is the intermolecular distance and is set to 7.2 Å as in the case of rubrene crystal. Although we have treated the model as a closed system, in our study the recurrence problem is not severe and *C*(*t*) in general rapidly decays to nearly zero, except when both *V* and Δ*V* are small. In such cases we resort to a more strict model with 10 modes in total for the convergence of *C*(*t*). Lying at the heart of our calculation is the evaluation of the current–current correlation function $C(t)=\left\langle \hat{j}(t)\hat{j}(0)\right\rangle$, which is achieved by the time-dependent MPS formalism^21,26,27^. In most of our simulations, the number of molecules in the periodic one-dimensional chain is 21 and the virtual bond dimension is 80. More details on the model with 9 intramolecular modes as well as numerical convergence check on system size and MPS parameters are included in Supplementary Table 1, Supplementary Fig. 1, and Supplementary Fig. 2.

### Carrier mobility

**Fig. 1:**
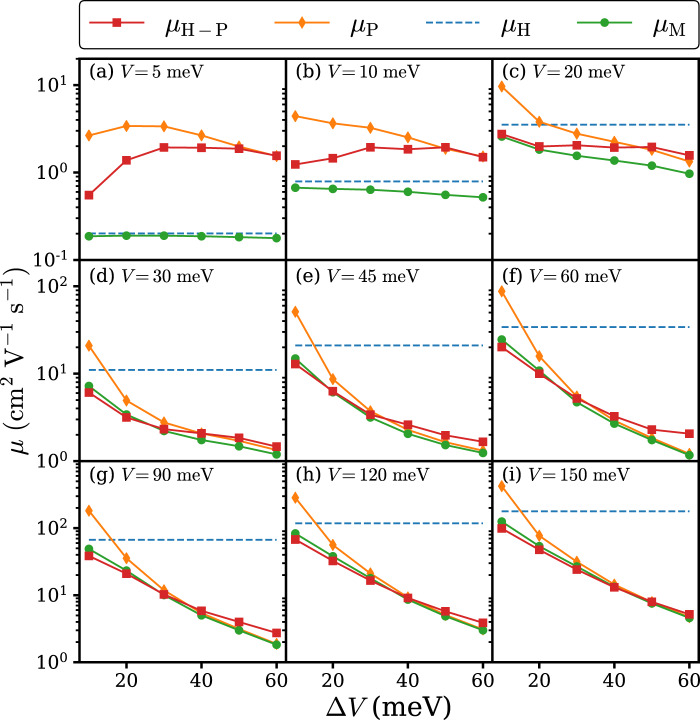
Carrier mobility at 300 K calculated based on the Holstein–Peierls model (*μ*_H-P_), Holstein model (*μ*_H_), Peierls model (*μ*_P_), and Matthiessen’s rule (*μ*_M_) with various transfer integral *V* and transfer integral fluctuation Δ*V*. From **a** to **i** the transfer integrals are 5, 10, 20, 30, 45, 60, 90, 120, and 150 meV respectively. Other parameters relevant to the carrier mobility such as local EPC constants are fixed with values taken from rubrene. The parameters for the Holstein (Peierls) model is the same as that of Holstein–Peierls model except that the nonlocal (local) EPC is left out.

Firstly, we analyze the role of local and nonlocal EPC on different parameter regimes by comparing the mobility calculated based on the Holstein–Peierls model (*μ*_H-P_) with the mobility calculated based on pure Holstein model (*μ*_H_) and pure Peierls model (*μ*_P_) at 300 K. We have also included the mobility derived from Matthiessen’s rule (1/*μ*_M_ = 1/*μ*_H_ + 1/*μ*_P_), presumably valid in the BL regime, because in the BL regime both local and nonlocal EPC can be considered as independent scattering sources and the total scattering rate of the wave-like electronic motion is the sum of both scattering rates. The overall results are shown in Fig. 1. When *V* = 5 meV, *μ*_H-P_ is generally higher than *μ*_H_, which implies that nonlocal EPC is beneficial to charge transport. This is considered to be a signature of the PA picture. However, this behavior quickly vanishes as *V* increases from 5 meV to 20 meV. At the intermediate range of *V*, e.g. from 45 meV to 120 meV, *μ*_H-P_ could be higher than *μ*_P_ for larger Δ*V*. That local EPC could enhance instead of reduce mobility is quite counter-intuitive. Such unusual behavior can be best understood by TL picture, in which the quantum coherent interference responsible for Anderson localization can be damaged or destroyed by local EPC as the dephasing noise. The mechanism is studied in detail by means of open system dynamics for systems with static disorder^37,38^. In addition, the “band width narrowing” caused by local EPC could allow the carrier to thermally access much delocalized states^17^. These lead to the local EPC enhanced mobility. Upon further increasing *V* to 150 meV, the TL scenario also becomes less significant. Instead, it is found that *μ*_M_ coincides with *μ*_H-P_ remarkably well, serving as a piece of evidence for band-like transport.

### Mean free path and optical conductivity

Although in the BL regime *μ*_M_ is expected to be a good approximation for *μ*_H-P_, *μ*_M_ ≈ *μ*_H-P_ alone is not a sufficient condition for band-like transport, and we additionally employ the Mott-Ioffe-Regel limit for the determination of the BL regime. The carrier mean free path *l*_mfp_ is estimated as *l*_mfp_ = *vτ* with the group velocity *v* and relaxation time *τ* evaluated by^39^:

5 $$v =\sqrt{\left\langle \hat{j}(0)\hat{j}(0)\right\rangle }/{e}_{0}\\ \tau = \int_{0}^{\infty }\left|\frac{{\rm{Re}}C(t)}{{\rm{Re}}C(0)}\right|{\mathrm{d}}t.$$

**Fig. 2:**
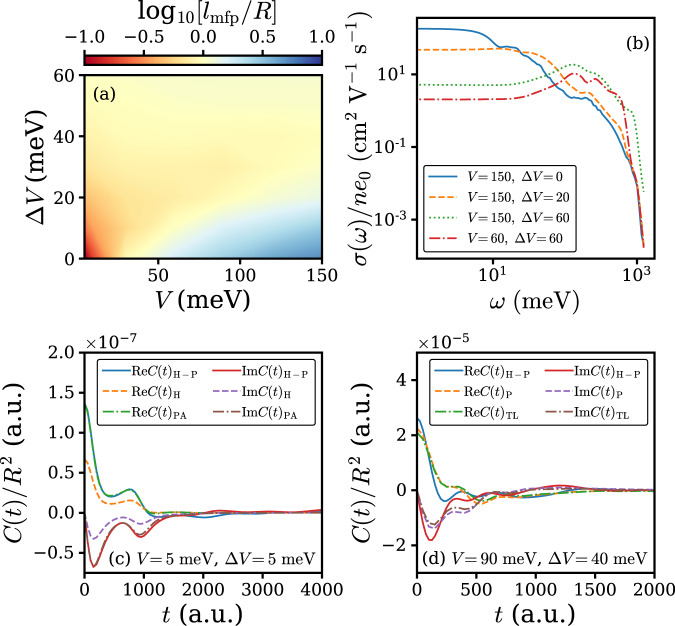
Further analysis of the transport regimes. **a** Carrier mean free path *l*_mfp_/*R* at 300 K for the Holstein-Peierls model at various transfer integral *V* and transfer integral fluctuation Δ*V*. In the blue region (bottom right) the carrier mean free path exceeds the lattice constant *R*. **b** Per carrier optical conductivity of the Holstein–Peierls model at various transfer integral *V* and transfer integral fluctuation Δ*V*. **c**, **d** Correlation functions obtained from our simulation with Holstein-Peierls model *C*(*t*)_H-P_, pure Holstein model *C*(*t*)_H_ and pure Peiels model *C*(*t*)_P_ as well as the correlation function obtained from phonon-assisted transport theory^24^*C*(*t*)_PA_ and transient localization theory^40^*C*(*t*)_TL_ for **c** *V* = 5 meV, Δ*V* = 5 meV and **d** *V* = 90 meV, Δ*V* = 40 meV.

And the calculated *l*_mfp_ in the (*V*, Δ*V*) plane at 300 K is shown in Fig. 2a. The overall tendency of *l*_mfp_ matches well with the carrier mobility of the Holstein–Peierls model *μ*_H-P_ in Fig. 1. The region where *l*_mfp_ > *R* is colored with blue in Fig. 2a and it lies within the large *V*, small Δ*V* limit, and in agreement with common perception. Another possible criteria for BL conduction is the appearance of Drude-like peak in the per carrier optical conductivity:

6 $$\frac{\sigma (\omega )}{n{e}_{0}}=\frac{1-{e}^{-\omega /{k}_{B}T}}{\omega }\int_{0}^{\infty }C(t){e}^{i\omega t}{\mathrm{d}}t$$

which is illustrated in Fig. 2b. In the case of *V* = 150 meV without nonlocal EPC, a broad Drude-like peak appears near *ω* = 0. Upon adding a small amount of nonlocal EPC, the Drude-like peak becomes invisible. However, the optical conductivity is still significantly different from the Δ*V* = 60 meV cases, in which a localization peak at *ω* ≈ 200 meV characteristic for the TL regime^18^ is present.

### Connection with semi-analytical theories

The PA regime and the TL regime can be further confirmed by semi-analytical results. In Fig. 2c we compare *C*(*t*)_H-P_ of our numerical simulation with *C*(*t*)_PA_ of phonon-assisted charge transport theory^24^:

7 $$\mu 	=\frac{{e}_{0}{R}^{2}}{{k}_{B}T}\int_{-\infty }^{\infty }[{V}^{2}+{({g}_{\theta ,{\rm{II}}}{\omega }_{m})}^{2}{{{\Phi }}}_{\theta }(t)]{e}^{-{{\Gamma }}(t)}{\mathrm{d}}t\\ {{\Gamma }}(t) 	=2\mathop{\sum}\limits_{m}{g}_{m,{\rm{I}}}^{2}[1+2{N}_{m}-{{{\Phi }}}_{m}(t)]+4{g}_{\theta ,{\rm{II}}}^{2}[1+2{N}_{\theta }-{{{\Phi }}}_{m}(t)]\\ {{{\Phi }}}_{m} 	=(1+{N}_{m}){e}^{-i{\omega }_{m}t}+{N}_{m}{e}^{i{\omega }_{m}t}\\ {N}_{m} 	=\frac{1}{{e}^{{\omega }_{m}/{k}_{B}T}-1}$$

The parameters are *V* = 5 meV and Δ*V* = 5 meV. For both real and imaginary part the two curves are in excellent agreement, and show significant increase with respect to the correlation function with only local EPC *C*(*t*)_H_. Thus we can confidently conclude that in this parameter regime the transport mechanism can be understood as phonon-assisted transport. We note that the derivation of Eq. (7) employs narrow-band approximation, which is valid in the small *V* limit. When *V* = 90 meV and Δ*V* = 40 meV shown in Fig. 2d, the correlation function given by TL theory with relaxation time approximation^40^ *C*(*t*)_TL_ is in agreement with our calculation based on pure Peierls model *C*(*t*)_P_. The observation implies that in this regime the TL theory can successfully account for the transport property of the pure Peierls model, from which the TL theory is derived. If the Holstein coupling is included, the correlation function *C*(*t*)_H-P_ exhibits significant difference from *C*(*t*)_P_ and *C*(*t*)_TL_, however, the integrated mobility turns out to be rather insensitive to Holstein coupling in this regime (Fig. 1g). We note that it is possible to integrate Holstein coupling in the transient localization theory if the intramolecular vibration frequency is much smaller than the transfer integral^33,41^, however such scheme is not employed in this work because in most cases *ω*_*m*_ is at the same order with *V*. We believe it is suitable to ascribe the *V* = 90 meV and Δ*V* = 40 meV case as TL, because although the TL theory with relaxation time approximation may not correctly produce the correlation function for realistic materials with Holstein coupling, the picture provided by the theory serves as a nice starting point for further analysis.

### Effect of local EPC strength

**Fig. 3:**
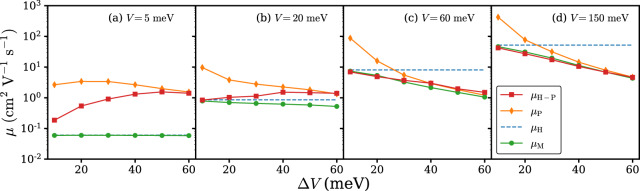
Carrier mobility at 300 K calculated based on the Holstein–Peierls model (*μ*_H-P_), Holstein model (*μ*_H_), Peierls model (*μ*_P_), and Matthiessen’s rule (*μ*_M_) with enlarged local EPC at various transfer integral *V* and transfer integral fluctuation Δ*V*. From **a**–**d** the transfer integrals are 5, 20, 60, and 150 meV respectively.

**Fig. 4:**
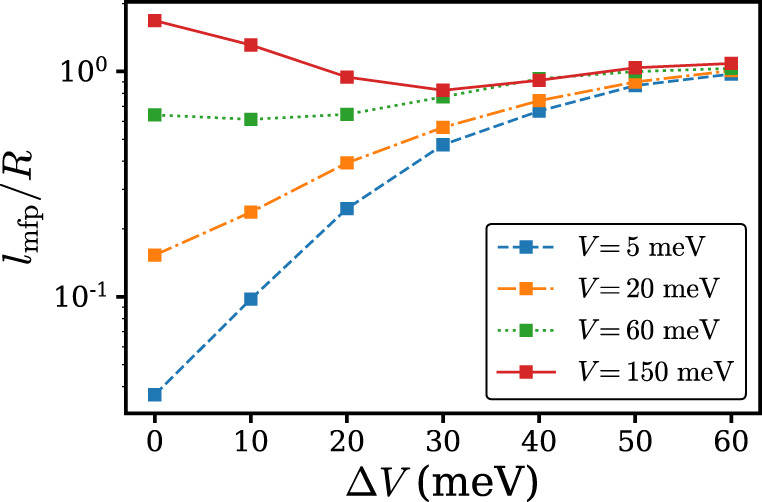
Carrier mean free path with enlarged local EPC. Carrier mean free path *l*_mfp_/*R* at 300 K for the Holstein–Peierls model with enlarged local EPC at various transfer integral *V* and transfer integral fluctuation Δ*V*.

In order to investigate how local EPC strength will affect the results in Fig. 1, we have further calculated the carrier mobility of the Holstein–Peierls model with stronger local EPC. More specifically, the values of *g*_*m*,I_ are multiplied by $\sqrt{2}$ so that the respective reorganization energies ${g}_{m,{\rm{I}}}^{2}{\omega }_{m}$ are doubled. The results are illustrated in Fig. 3. In the small *V* limit shown in Fig. 3a, the PA mechanism prevails, in agreement with the results in Fig. 1. However, from Fig. 3b it can be seen that with enlarged local EPC strength the PA picture remains valid even if *V* becomes as large as 20 meV, in contrast to the *V* = 20 meV results presented in Fig. 1, indicating the expansion of the PA region. Accordingly, the TL region is diminished, as can be inferred from Fig. 3c, d by noting that the parameter space in which *μ*_H-P_ ≥ *μ*_P_ is satisfied is smaller than that of Fig. 1. In Fig. 4 we show the carrier mean free path *l*_mfp_/*R* with increased local EPC strength. When *V* is relatively large and Δ*V* is relatively small, namely in the BL regime, *l*_mfp_/*R* with increased local EPC strength is generally smaller than that of the original local EPC strength. This observation implies that the BL region in the (*V*, Δ*V*) plane moves toward the even larger *V* area (*V* > 150 meV). Our findings are in agreement with physical instinct because in the large local EPC limit the hopping mechanism dominates and Eq. (7) is a good approximation for mobility. Another factor that might affect charge transport is the distribution of intramolecular vibration frequencies with fixed total reorganization energy. The problem is equivalent to the isotope effect problem and recent studies on the rubrene molecule concluded negative isotope effect^6,9,21^.

### One-particle spectral function

**Fig. 5:**
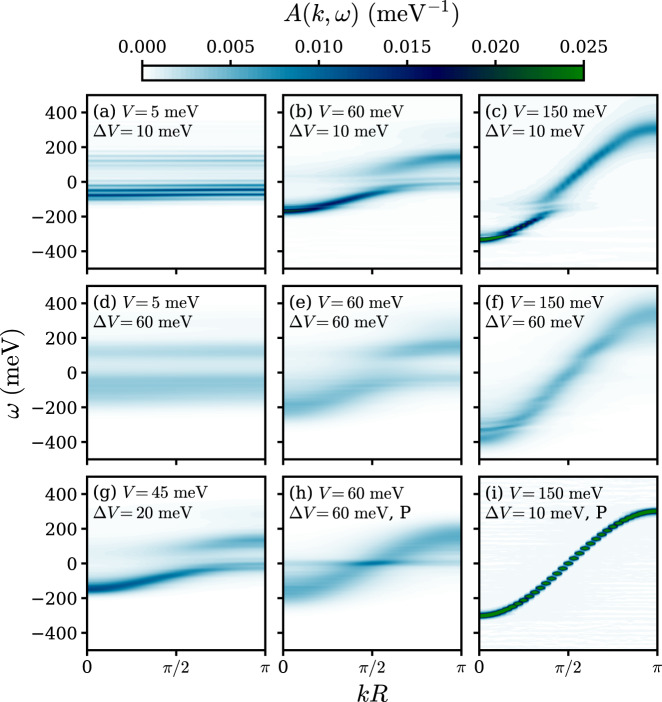
Spectral function for the Holstein-Peierls model and pure Peierls model. Spectral function at 300 K for the Holstein–Peierls model (**a**–**g**) and the pure Peierls model (**h**, **i**). Panel **h** and **i** has the same parameter as panel **e** and **c** respectively except that in Panel **h** and **i** the local EPC is left out.

To further analyze the charge transport properties in the regimes implied by Figs. 1 and 2, we calculated the momentum resolved one-particle spectral function:

8 $$A(k,\omega )=\frac{1}{N\pi }\mathop{\sum }\limits_{mn}^{N}{e}^{ikR(m-n)}\int_{0}^{\infty }\left\langle {c}_{m}(t){c}_{n}^{\dagger }(0)\right\rangle {e}^{i\omega t}{\mathrm{d}}t$$

for nine sets of representative parameters at 300 K in Fig. 5. A Lorentzian broadening with *η* = 5 meV is applied for a smooth spectra. When *V* = 5 meV and Δ*V* = 10 meV (Fig. 5a), the spectral function exhibits dispersionless bound states separated by the intramolecular vibration frequencies *ω*_*m*_, which marks the formation of small polaron. On the contrary, when *V* = 150 meV and Δ*V* = 10 meV (Fig. 5c) an intense quasiparticle peak is observed near *k* = 0 and the overall shape of the spectra resembles the dispersion of free electron $E(k)=-2V\cos kR$. In the TL regime represented by *V* = 60 meV and Δ*V* = 60 meV (Fig. 5e) the signature of either small polaron or delocalized state is almost completely smeared out. In combination with the limited carrier mean free path in this regime, it can be deduced that the charge carrier is localized by nonlocal EPC instead of local EPC. The same “blurred” spectral function is observed for other sets of typical parameters in the TL regime (Fig. 5d, f). With moderate *V* and Δ*V* shown in Fig. 5g, the spectral function exhibits none of the typical features described above. Namely, although the spectral function does not manifest the formation of small polaron or delocalized states, the peak intensity is still strong enough to be discernible from the TL regimes cases (Fig. 5d–f). In the absence of the local EPC (Fig. 5h, i), the quasiparticle peak has more intensity, implying the disruption of quantum coherent with the addition of local EPC. Combined with the mobility data shown in Fig. 1, it can be inferred that in the TL regime, the effect of the disruption is to alleviate the localization caused by nonlocal EPC, leading to increased mobility (Fig. 5e, h), while in the BL regime, on the contrary, the disruption scatters charge carrier and reduces mobility (Fig. 5c, i). The horizontal peak at the center of the band in Fig. 5h is a result of the pure nonlocal EPC in the Peierls model and is expected to vanish upon the inclusion of infinitesimal local EPC^42^.

### The isotropy effect

In a number of recent works it is established that dimensionality plays an indispensable role in the charge transport process, especially when dynamic disorder is taken into consideration^8,13,43^. To study the isotropy effect beyond the one-dimensional model, we employ a quasi-two-dimensional ladder Holstein–Peierls Hamiltonian:

9 $$\hat{H}	= \, {\hat{H}}_{{\rm{e}}}+{\hat{H}}_{{\rm{ph}}}+{\hat{H}}_{{\rm{e-ph}}}\\ {\hat{H}}_{{\rm{e}}}	= -{V}_{1}\mathop{\sum}\limits_{l=1,2}\mathop{\sum}\limits_{n}({c}_{l,n+1}^{\dagger }{c}_{l,n}+{c}_{l,n}^{\dagger }{c}_{l,n+1})-{V}_{2}\mathop{\sum}\limits_{n}({c}_{0,n}^{\dagger }{c}_{1,n}+{c}_{1,n}^{\dagger }{c}_{0,n})\\ {\hat{H}}_{{\rm{ph}}}	= \mathop{\sum}\limits_{l,n,m}{\omega }_{m}{b}_{l,n,m}^{\dagger }{b}_{l,n,m}+\mathop{\sum}\limits_{l,n}{\omega }_{\theta }{b}_{l,n,\theta }^{\dagger }{b}_{l,n,\theta }\\ {\hat{H}}_{{\rm{e-ph}}}	= \mathop{\sum}\limits_{l,n,m}{g}_{m,{\rm{I}}}{\omega }_{m}({b}_{l,n,m}^{\dagger }+{b}_{l,n,m}){c}_{l,n}^{\dagger }{c}_{l,n}\\ 	+\mathop{\sum}\limits_{l,n}{g}_{\theta ,{\rm{II}}}{\omega }_{\theta }({b}_{l,n,\theta }^{\dagger }+{b}_{l,n,\theta })({c}_{l,n+1}^{\dagger }{c}_{l,n}+{c}_{l,n}^{\dagger }{c}_{l,n+1})$$

here *V*_1_ and *V*_2_ represent the electronic coupling at the high-mobility direction and the low-mobility direction respectively. The intermolecular vibration at the *V*_2_ direction is neglected for simplicity. The setup, while still approximate compared to a full-fledged two-dimensional model, is believed to be reasonable for anisotropic materials (*V*_2_ ≪ *V*_1_) and can at least partially capture the dimensionality effect. In Fig. 6 we present the computed correlation function *C*(*t*) and mobility *μ* based on the model for several typical values of *V*_1_ and Δ*V*_1_. In the hopping limit shown in Fig. 6a, e, it is found that carrier mobility is irrelevant to the isotropy effect, because in this limit *V*_2_ does not affect the hopping process at *V*_1_ direction. In the phonon-assisted transport regime shown in Fig. 6b, f, *μ* is rather insensitive to isotropy effect. In the band-like regime shown in Fig. 6c, g, we find that isotropy effect tends to slightly increase mobility. Lastly, in the transient localization regime shown in Fig. 6d, h, it is observed that carrier mobility is susceptible to the isotropy effect. By increasing *V*_2_ from 0 to 0.2*V*_1_, the mobility increases by ~40%. Such increase may appear difficult to understand as *C*(*t*) in Fig. 6d does not seem to vary much. This is because transient localization implies that during the integration of *C*(*t*) the positive and the negative part of *C*(*t*) are canceled out, and thus minor changes in *C*(*t*) will result in a big difference in mobility. Our result is generally in agreement with previous literatures^8,13^. Based on these findings we can conclude that when the isotropy of the system is increased, the band region tend to expand while the transient localization regime would tend to diminish^18^. Physically, the first conclusion can be understood by enlarged bandwidth in two dimension and the second conclusion can be understood by considering that Anderson localization length for two dimension is larger than that in one dimension^44^.

**Fig. 6:**
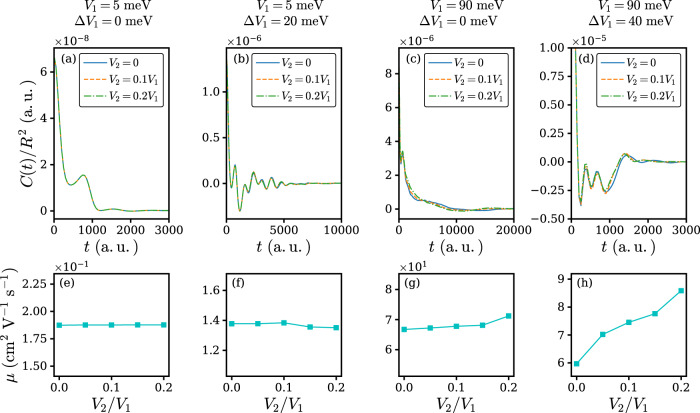
The isotropy effect for several typical values of *V*_1_ and Δ*V*_1_. **a**–**d** are the correlation functions *C*(*t*) and **e**–**h** are the corresponding mobilities *μ*. For **a** and **e**, *V*_1_ = 5 meV and Δ*V*_1_ = 0 meV; For **b** and **f**, *V*_1_ = 5 meV and Δ*V*_1_ = 20 meV; For **c** and **g**, *V*_1_ = 90 meV and Δ*V*_1_ = 0 meV; For **d** and **h**, *V*_1_ = 90 meV and Δ*V*_1_ = 40 meV.

### General charge transport regime diagram

**Fig. 7:**
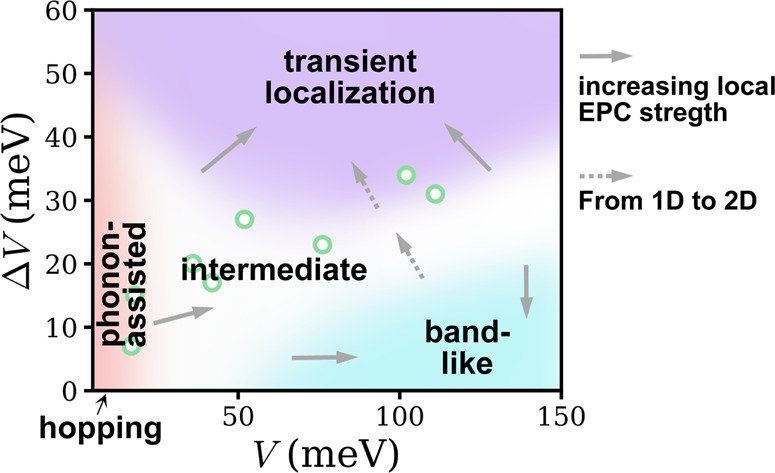
A schematic “regime diagram” showing the phonon-assisted transport (PA) regime, transient localization (TL) regime, band-like (BL) regime, and intermediate regime on the (*V*, Δ*V*) plane for the carrier mobility of the Holstein–Peierls model. The hopping regime is achieved in the Δ*V* = 0 limit of the PA regime. Gray solid arrows show qualitatively the shift of the boundaries when local EPC increases. Gray dashed arrows show qualitatively the shift of the boundaries when transport network changes from one dimension to quasi-two-dimension or equivalently when electronic coupling isotropy increases. The green dots represent the *V* and Δ*V* of several common organic semiconductors.

Based on the EPC effect on the carrier mobility, the mean free path, the optical conductivity, and the one-particle spectral function, we are able to sketch a schematic “regime diagram” for the charge transport mechanisms as shown in Fig. 7. The PA regime is determined by *μ*_H-P_ > *μ*_H_, short *l*_mfp_, *C*(*t*)_H-P_ ≈ *C*(*t*)_PA_ and narrow bound state states in the spectral function. The TL regime is determined by *μ*_H-P_ ≥ *μ*_P_, intermediate *l*_mfp_, localization peak in optical conductivity, *C*(*t*)_P_ ≈ *C*(*t*)_TL_ and a “smeared out” spectral function. The BL regime is determined by *μ*_H-P_ ≈ *μ*_M_, large *l*_mfp_, Drude-like peak in optical conductivity and sharp quasiparticle peak in the spectral function. In Fig. 7 we use *μ*_H-P_ > *μ*_H_, *μ*_H-P_ ≥ *μ*_P_ and *l*_mfp_ > *R* for the boundaries of the PA regime, TL regime, and BL regime respectively, and using other indicators such as the appearance of Drude-like peak for the BL regime may shift the boundaries to some extent but the general picture remains intact. On this (*V*, Δ*V*) plane we are also able to identify an “intermediate” regime that lies among the PA regime, TL regime, and BL regime. In this regime, *μ*_H-P_ is significantly lower than both *μ*_H_ and *μ*_P_, and the carrier mean free path is still less than the lattice constant, forbidding the band description. In fact, for the pure Holstein model case (Δ*V* = 0), the intermediate regime simply degenerates into the canonical hopping-band crossover. The crossover from the BL regime to the TL regime has also been reported by introducing transient localization correction to band transport^18^. The gray solid arrows and gray dashed arrows in Fig. 7 indicate the shift of the boundaries upon increasing local EPC and increasing electronic coupling isotropy respectively, based on Figs. 3, 4 and 6. To provide a rough intuition of the distribution of parameters for realistic organic semiconductors on this (*V*, Δ*V*) plane, in Fig. 7 we have also marked the value of *V* and Δ*V* for several common organic semiconductors using reported values from recent literature^32^. These materials are pMSB, pyrene, naphthalene, perylene, anthracene, DATT, rubrene, and pentacene from left to right. We note that the colors at the location of the markers do not represent a prediction of the charge transport mechanism for the corresponding materials because the materials do not necessarily share the same local EPC coupling strength, transport network and intermolecular vibration frequency with the parameters used in this work.

## Discussion

In this work, we present a nearly exact theoretical study of the carrier mobility in Holstein–Peierls model with parameters relevant to organic semiconductors. By carefully investigating the effect of both local and nonlocal EPCs on the carrier mobility *μ*, mean free path *l*_mfp_, per carrier optical conductivity $\frac{\sigma (\omega )}{n{e}_{0}}$, and one-particle spectral function *A*(*k*, *ω*), we are able to identify the PA regime, TL regime and BL regime on the (*V*, Δ*V*) plane. The PA regime features *μ*_H-P_ > *μ*_H_, short *l*_mfp_, and narrow bound states in the spectral function. The TL regime features *μ*_H-P_ ≥ *μ*_P_, intermediate *l*_mfp_, localization peak in optical conductivity and a “smeared out” spectral function. And the BL regime features *μ*_H-P_ ≈ *μ*_M_, large *l*_mfp_, Drude-like peak in optical conductivity and sharp quasiparticle peak in the spectral function. The semiclassical Marcus hopping regime is found to be around the corner of small *V* and Δ*V*. Furthermore, some of the parameters in the (*V*, Δ*V*) plane are recognized to lie in an intermediate regime that does not exhibit the typical features described above, and this regime can be considered as a generalization of the hopping-band crossover regime in the Holstein model. We find that when increasing local EPC strength, the PA regime will expand while the TL and BL regime will diminish. When going from one dimension to quasi-two-dimension, the TL regime will diminish and the BL regime will expand. It should be noted that the localization effect due to static disorder is not considered here, which deserves further investigation.

## Methods

### Matrix product states

The evaluation of the current–current correlation function $C(t)=\left\langle \hat{j}(t)\hat{j}(0)\right\rangle$ is performed by time-dependent matrix product states through imaginary and real time propagation. The matrix product states method represent the wavefunction of many-body system as the product of a series of matrices^26^:

10 $$\left|{{\Psi }}\right\rangle =\mathop{\sum}\limits_{\{a\},\{\sigma \}}{A}_{{a}_{1}}^{{\sigma }_{1}}{A}_{{a}_{1}{a}_{2}}^{{\sigma }_{2}}\cdots {A}_{{a}_{N-1}}^{{\sigma }_{N}}\left|{\sigma }_{1}{\sigma }_{2}\cdots {\sigma }_{N}\right\rangle$$

$\left|{\sigma }_{i}\right\rangle$ is the basis for each degree of freedom. ${A}_{{a}_{i-1}{a}_{i}}^{{\sigma }_{i}}$ are matrices in the chain connected by indices *a*_*i*_. {⋅} in the summation represents the contraction of the respective connected indices, and *N* is the total number of degrees of freedom (DOFs) in the system. The dimension of *a*_*i*_ is called (virtual) bond dimension, while the dimension of *σ*_*i*_ is called physical bond dimension. In principle, the time-dependent algorithms for MPS^27^ is able to solve the time-dependent Schrödinger equation in an exact manner if the bond dimension is infinite. In practice, the accuracy of the method can be systematically improved by using a larger bond dimension, until convergence of interested physical observables within arbitrary convergence criteria.

### Finite temperature algorithm

The finite temperature effect is taken into account through thermal field dynamics, also known as the purification method^26,45^. The thermal equilibrium density matrix of any mixed state in physical space *P* can be expressed as a partial trace over an enlarged Hilbert space *P* ⊗ *Q*, where *Q* is an auxiliary space chosen to be a copy of *P*. The thermal equilibrium density operator can then expressed as a partial trace of the pure state Ψ_*β*_ in the enlarged Hilbert space over the *Q* space:

11 $${\hat{\rho }}_{\beta }=\frac{{e}^{-\beta \hat{H}}}{Z}=\frac{{{\rm{Tr}}}_{Q}|{{{\Psi }}}_{\beta }\rangle \langle {{{\Psi }}}_{\beta }|}{{{\rm{Tr}}}_{PQ}|{{{\Psi }}}_{\beta }\rangle \langle {{{\Psi }}}_{\beta }|}$$

and the pure state $|{{{\Psi }}}_{\beta }\rangle$ represented as an MPS is obtained by the imaginary time propagation from the locally maximally entangled state $\left|I\right\rangle ={\sum }_{i}{\left|i\right\rangle }_{P}{\left|i\right\rangle }_{Q}$ to *β*/2 in the one electron manifold:

12 $$|{{{\Psi }}}_{\beta }\rangle ={e}^{-\beta \hat{H}/2}|I\rangle .$$

To calculate *C*(*t*), $|{{{\Psi }}}_{\beta }\rangle$, and $\hat{j}(0)|{{{\Psi }}}_{\beta }\rangle$ are propagated in real time to obtain ${e}^{-i\hat{H}t}|{{{\Psi }}}_{\beta }\rangle$ and ${e}^{-i\hat{H}t}\hat{j}(0)|{{{\Psi }}}_{\beta }\rangle$ and then *C*(*t*) is calculated by:

13 $$C(t)=\langle {{{\Psi }}}_{\beta }|{e}^{i\hat{H}t}\hat{j}(0){e}^{-i\hat{H}t}\hat{j}(0)|{{{\Psi }}}_{\beta }\rangle /Z.$$

Here the current operator $\hat{j}(0)$ is represented as an MPO and inner-product for $|{{{\Psi }}}_{\beta }\rangle$ includes tracing over both *P* space and *Q* space. The construction of the MPOs is performed in an automatic and optimal fashion through our recently proposed algorithm^46^. Note that different from the simulation of diffusion dynamics, the initial state of the formulation does not require electronic excitation from the zero electron manifold. In principle, both imaginary and real time propagation can be carried out by any time evolution methods available to matrix product states^27^. In this work, we use the time-dependent variational principle based projector splitting time evolution scheme^47,48^, which is found to be relatively efficient and accurate combined with graphic processing unit (GPU) in our recent work^49^.

## Supplementary information

Suplementary Information

## Data Availability

The data generated in this study has been deposited in Zenodo with DOI 10.5281/zenodo.5009584.

## Code availability

The computer code for the MPS algorithms used in this work is available publicly via https://github.com/shuaigroup/Renormalizer^50^.

## References

[1] Podzorov V (2004). Intrinsic charge transport on the surface of organic semiconductors. Phys. Rev. Lett..

[2] Podzorov V, Menard E, Rogers JA, Gershenson ME (2005). Hall effect in the accumulation layers on the surface of organic semiconductors. Phys. Rev. Lett..

[3] Mitsui C (2014). High-performance solution-processable n-shaped organic semiconducting materials with stabilized crystal phase. Adv. Mater..

[4] Brédas JL, Calbert JP, da Silva Filho DA, Cornil J (2002). Organic semiconductors: a theoretical characterization of the basic parameters governing charge transport. Proc. Natl Acad. Sci. USA.

[5] Nan G, Yang X, Wang L, Shuai Z, Zhao Y (2009). Nuclear tunneling effects of charge transport in rubrene, tetracene, and pentacene. Phys. Rev. B.

[6] Jiang Y (2016). Nuclear quantum tunnelling and carrier delocalization effects to bridge the gap between hopping and bandlike behaviors in organic semiconductors. Nanoscale Horiz..

[7] Jiang Y, Peng Q, Geng H, Ma H, Shuai Z (2015). Negative isotope effect for charge transport in acenes and derivatives - a theoretical conclusion. J. Phys. Chem. Lett..

[8] Wang L, Li Q, Shuai Z, Chen L, Shi Q (2010). Multiscale study of charge mobility of organic semiconductor with dynamic disorders. Phys. Chem. Chem. Phys..

[9] Ren X (2017). Negative isotope effect on field-effect hole transport in fully substituted ^13^C-ubrene. Adv. Electron. Mater..

[10] Platt AD, Kendrick MJ, Loth M, Anthony JE, Ostroverkhova O (2011). Temperature dependence of exciton and charge carrier dynamics in organic thin films. Phys. Rev. B.

[11] Fratini S, Ciuchi S (2009). Bandlike motion and mobility saturation in organic molecular semiconductors. Phys. Rev. Lett..

[12] Fratini S, Mayou D, Ciuchi S (2016). The transient localization scenario for charge transport in crystalline organic materials. Adv. Funct. Mater..

[13] Fratini S, Ciuchi S, Mayou D, de Laissardière GT, Troisi A (2017). A map of high-mobility molecular semiconductors. Nat. Mater..

[14] Schweicher G (2019). Chasing the “killer” phonon mode for the rational design of low-disorder, high-mobility molecular semiconductors. Adv. Mater..

[15] Kubo T (2016). Suppressing molecular vibrations in organic semiconductors by inducing strain. Nat. Commun..

[16] Illig S (2016). Reducing dynamic disorder in small-molecule organic semiconductors by suppressing large-amplitude thermal motions. Nat. Commun..

[17] Fetherolf JH, Golež D, Berkelbach TC (2020). A unification of the Holstein polaron and dynamic disorder pictures of charge transport in organic crystals. Phys. Rev. X.

[18] Fratini S, Ciuchi S (2020). Dynamical localization corrections to band transport. Phys. Rev. Res..

[19] Holstein T (1959). Studies of polaron motion: Part I. the molecular-crystal model. Ann. Phys..

[20] Mishchenko AS, Nagaosa N, De Filippis G, de Candia A, Cataudella V (2015). Mobility of Holstein polaron at finite temperature: An unbiased approach. Phys. Rev. Lett..

[21] Li W, Ren J, Shuai Z (2020). Finite-temperature TD-DMRG for the carrier mobility of organic semiconductors. J. Phys. Chem. Lett..

[22] Munn R, Silbey R (1985). Theory of electronic transport in molecular crystals. III. diffusion coefficient incorporating nonlocal linear electron–phonon coupling. J. Chem. Phys..

[23] Hannewald K (2004). Theory of polaron bandwidth narrowing in organic molecular crystals. Phys. Rev. B.

[24] Hannewald K, Bobbert PA (2004). Anisotropy effects in phonon-assisted charge-carrier transport in organic molecular crystals. Phys. Rev. B.

[25] Zhang W, Zhong X, Zhao Y (2012). Electron mobilities of n-type organic semiconductors from time-dependent wavepacket diffusion method: Pentacenequinone derivatives. J. Phys. Chem. A.

[26] Schollwöck U (2011). The density-matrix renormalization group in the age of matrix product states. Ann. Phys..

[27] Paeckel S (2019). Time-evolution methods for matrix-product states. Ann. Phys..

[28] Coropceanu V, Sánchez-Carrera RS, Paramonov P, Day GM, Brédas J-L (2009). Interaction of charge carriers with lattice vibrations in organic molecular semiconductors: naphthalene as a case study. J. Phys. Chem. C.

[29] Ruggiero MT, Ciuchi S, Fratini S, D’Avino G (2019). Electronic structure, electron-phonon coupling, and charge transport in crystalline rubrene under mechanical strain. J. Phys. Chem. C.

[30] Bussolotti F (2017). Hole-phonon coupling effect on the band dispersion of organic molecular semiconductors. Nat. Commun..

[31] Landi A, Troisi A (2018). Rapid evaluation of dynamic electronic disorder in molecular semiconductors. J. Phys. Chem. C.

[32] Giannini S (2019). Quantum localization and delocalization of charge carriers in organic semiconducting crystals. Nat. Commun..

[33] Fratini S, Nikolka M, Salleo A, Schweicher G, Sirringhaus H (2020). Charge transport in high-mobility conjugated polymers and molecular semiconductors. Nat. Mater..

[34] Troisi A (2007). Prediction of the absolute charge mobility of molecular semiconductors: the case of rubrene. Adv. Mater..

[35] Bässler H (1993). Charge transport in disordered organic photoconductors a monte carlo simulation study. Phys. Status Solidi (b).

[36] Mahan, G. D. *Many-Particle Physics* (Springer, 2000).

[37] Moix JM, Khasin M, Cao J (2013). Coherent quantum transport in disordered systems: I. the influence of dephasing on the transport properties and absorption spectra on one-dimensional systems. New J. Phys..

[38] Lee CK, Moix J, Cao J (2015). Coherent quantum transport in disordered systems: a unified polaron treatment of hopping and band-like transport. J. Chem. Phys..

[39] Prodanović N, Vukmirović N (2019). Charge carrier mobility in systems with local electron-phonon interaction. Phys. Rev. B.

[40] Ciuchi S, Fratini S, Mayou D (2011). Transient localization in crystalline organic semiconductors. Phys. Rev. B.

[41] Nematiaram T, Padula D, Landi A, Troisi A (2020). On the largest possible mobility of molecular semiconductors and how to achieve it. Adv. Funct. Mater..

[42] Theodorou G, Cohen MH (1976). Extended states in a one-demensional system with off-diagonal disorder. Phys. Rev. B.

[43] Sharma A, van Oost FWA, Kemerink M, Bobbert PA (2012). Dimensionality of charge transport in organic field-effect transistors. Phys. Rev. B.

[44] Lee PA, Ramakrishnan TV (1985). Disordered electronic systems. Rev. Mod. Phys..

[45] Feiguin AE, White SR (2005). Finite-temperature density matrix renormalization using an enlarged Hilbert space. Phys. Rev. B.

[46] Ren J, Li W, Jiang T, Shuai Z (2020). A general automatic method for optimal construction of matrix product operators using bipartite graph theory. J. Chem. Phys..

[47] Haegeman J, Cirac JI, Osborne TJ, Verschelde H, Verstraete F (2011). Time-dependent variational principle for quantum lattices. Phys. Rev. Lett..

[48] Haegeman J, Lubich C, Oseledets I, Vandereycken B, Verstraete F (2016). Unifying time evolution and optimization with matrix product states. Phys. Rev. B.

[49] Li W, Ren J, Shuai Z (2020). Numerical assessment for accuracy and GPU acceleration of TD-DMRG time evolution schemes. J. Chem. Phys..

[50] Ren, J., Li, W. & Jiang, T. A general charge transport picture for organic semiconductors with nonlocal electron-phonon couplings. *Renormalizer*10.5281/zenodo.4966913 (2021). 10.1038/s41467-021-24520-y PMC8275621 34253724

