# Supplementary material for: A general charge transport picture for organic semiconductors with nonlocal electron-phonon couplings
Source: Nat Commun. 2021 Jul 12;12:4260. doi: 10.1038/s41467-021-24520-y (PMC8275621; doi:10.1038/s41467-021-24520-y)
Supplement: Supplementary file 1 — Suplementary Information [file 41467_2021_24520_MOESM1_ESM.pdf]

Supporting Information for "A General Charge Transport Picture for Organic Semiconductors with Nonlocal Electron-Phonon Couplings"

Weitang Li, Jiajun Ren, and Zhigang Shuai[a)]

MOE Key Laboratory of Organic OptoElectronics and Molecular Engineering, Department of Chemistry, Tsinghua University, Beijing 100084, People's Republic of China

[a)]Electronic mail: zgshuai@tsinghua.edu.cn

I. THE MATRIX PRODUCT STATES PARAMETERS

In the following we present details on our MPS time evolution parameters and respective numerical convergence verification. The calculation of $C(t)$ involves three sets of time evolution: the imaginary time evolution for Ψ_β, real time evolution for $e^{-i\hat{H}t}|\Psi_\beta\rangle$ and real time evolution for $e^{-i\hat{H}t}\hat{j}(0)|\Psi_\beta\rangle$. For efficient calculation, we use different bond dimensions for the three time evolutions. More specifically, time evolutions for Ψ_β and $e^{-i\hat{H}t}|\Psi_\beta\rangle$ share the same bond dimension iM, and the time evolution for $e^{-i\hat{H}t}\hat{j}(0)|\Psi_\beta\rangle$ has the bond dimension M. Other important parameters relevant to the time evolution include the number of molecules in the periodic one dimensional chain N, the number of truncated harmonic oscillator eigenbasis for the intermolecular vibration l, and time evolution step dt. For most of our calculations, iM, M, N, l and dt are set to 64, 80, 21, 40, 50 respectively, and numerical convergence in the large transfer integral $V = 150$ meV and strong nonlocal EPC strength $\Delta V = 60$ meV limit is shown in Fig. 1. This is considered to be a set of challenging parameter due to the strong EPC and non-trivial physical behavior as discussed in the main text.

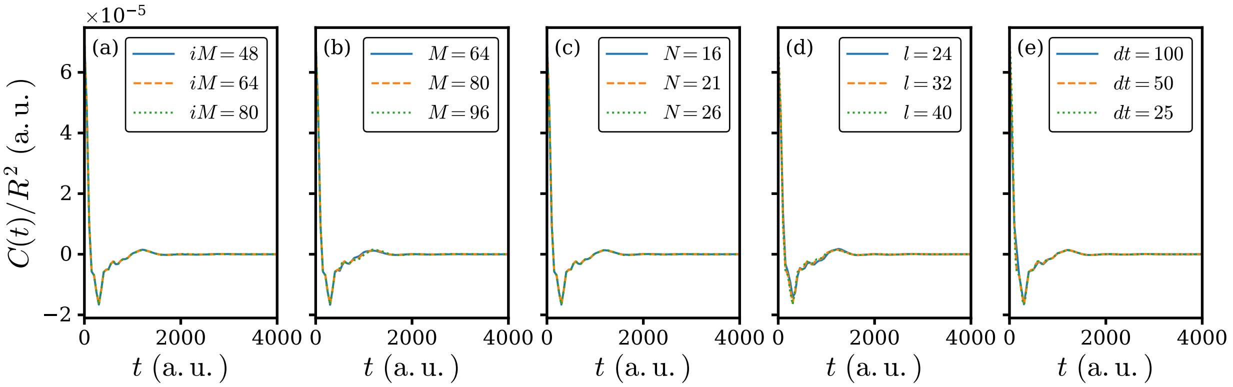

FIG. 1. Numeric convergence check for $C(t)$ of the Holstein-Peierls model in the $V = 150$ meV and $\Delta V =$ 60 meV case. From (a) to (e) the interested MPS parameter is imaginary time evolution bond dimension iM, real time evolution bond dimension M, system size N, number of intermolecular vibration basis l and time evolution step dt respectively.

We note, however, that a fixed set of MPS parameters is not sufficient for converged result over the whole $(V, \Delta V)$ plane explored in the main text, and we in fact adjust the MPS parameters accordingly to ensure that nearly exact result is obtained. We show in Fig. 2 the numerical convergence check for the pure Holstein model at $V = 150$ meV. In this regime, $C(t)$ decays at a slower

rate, requiring longer evolution time and consequently larger virtual bond dimension. Besides, the high mobility also demands larger system size in order to eliminate the finite size effect. So in the bottom-right corner of the $(V, \Delta V)$ plane we have carefully checked M and N and use values larger than 80 and 21 for M and N when necessary.

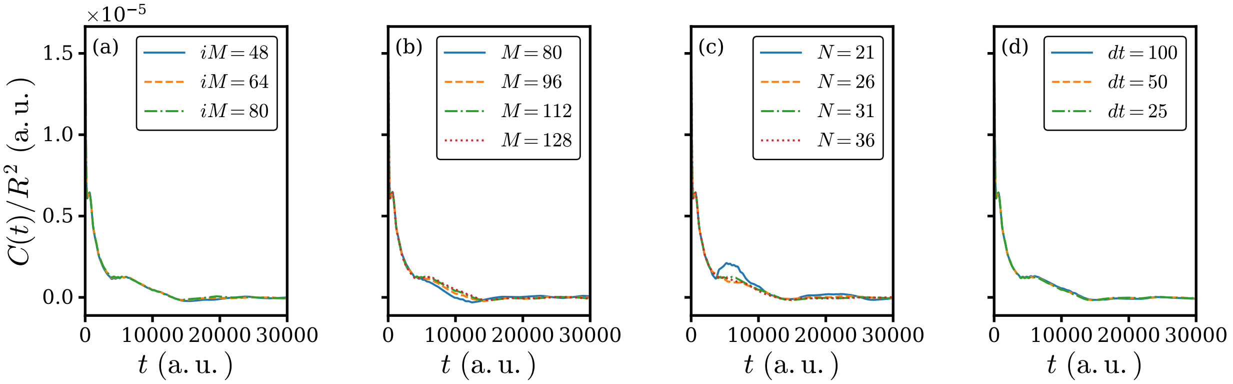

FIG. 2. Numeric convergence check for $C(t)$ of the pure Holstein model in the $V = 150$ meV case. From (a) to (d) the interested MPS parameter is imaginary time evolution bond dimension iM, real time evolution bond dimension M, system size N and time evolution step dt respectively.

II. BENCHMARK OF THE ONE-PARTICLE SPECTRAL FUNCTION

The evaluation of the one-particle spectral function $A(k, \omega)$ further takes advantage of the fact that the thermal equilibrium state is a zero-electron state. In this case, the thermal field dynamics algorithm can be reformulated to reduce computational cost[1,2]. For the calculation of $A(k, \omega)$ we use a system size of 64 and virtual bond dimension of 64. We benchmark our algorithm on a pure Holstein model with a single vibration mode at zero temperature, and our result (Fig. 3) is in quantitative agreement with exact diagonalization method[3].

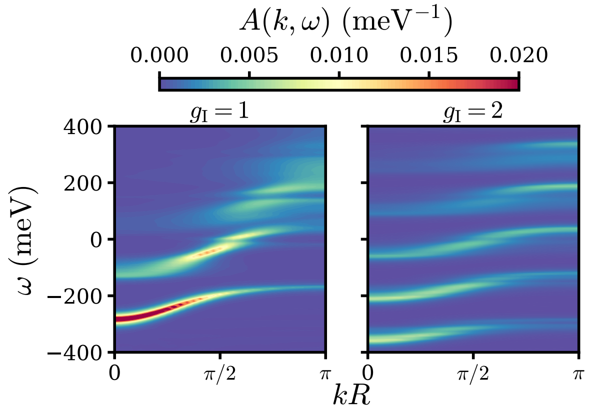

FIG. 3. Spectral function at zero temperature for the single-mode Holstein model. The parameters are: $V = 100$ meV, $\omega = 150$ meV and a Lorentzian broadening with $\eta = 10$ meV.

In Fig. 4 we compare the spectral function obtained from our simulation with the experimental angle resolved ultraviolet photoemission spectra(ARUPS) of rubrene, one of the best crystalline organic semiconductors[4]. The *ab initio* electronic coupling parameters for the simulation are $V = 90$ meV and $\Delta V = 30$ meV, for the rubrene crystal. In order to compare with the experimental ARUPS spectra of rubrene we have flipped the simulated spectral function along the y axis because of electron-hole symmetry. Fig. 4 shows that the theoretical prediction is in decent agreement with experimental observation in terms of bandwidth, subband splitting as well as the appearance of the kink. The result indicates that the MPS approach is reliable and the one-dimensional approximation in our model is valid at least for highly anisotropic organic semiconductors.

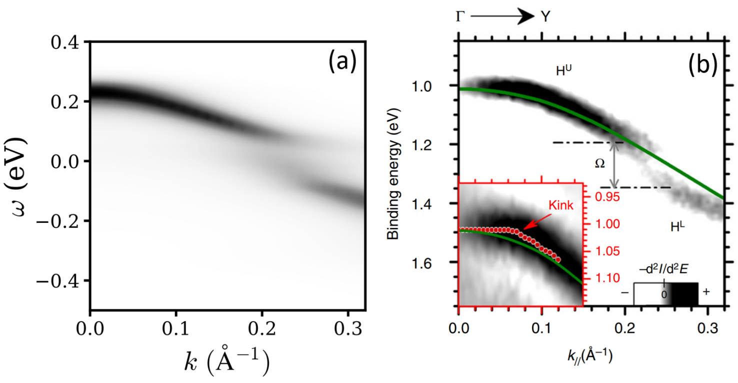

FIG. 4. Comparison between (a) simulated spectral function for rubrene and (b) experimental second derivative ARUPS spectra for rubrene[4] (licensed under Creative Commons Attribution 4.0 International License). The simulation parameters are $V = 90$ meV and $\Delta V = 30$ meV. Electron-phonon coupling strengths and lattice constants are specified in the main text.

III. THE NUMBER OF INTRAMOLECULAR VIBRATION MODES

Organic molecules usually contain dozens of atoms and it is common for more than 10 vibrational modes to contribute to local EPC. For simplicity, theoretical studies often reduce the modes into one effective mode[3,5,6]. However, by taking this approach, the correlation function $C(t)$ typically exhibits spurious long correlation time which is generally not possible for realistic material. In such cases, an artificial and sometimes arbitrary broadening is usually applied to $C(t)$ in order to mimic realistic world and determine an absolute value for mobility. In the main text most of our results are reported with a 4-(intramolecular-)mode model and $C(t)$ generally rapidly decays to zero. But in cases of small V and ΔV we have to further resort to a 9-(intramolecular-)mode model with vibration energy and EPC constant listed in Table I to avoid artificial recurrence. Starting from more than 40 vibrational modes that contribute to local EPC in rubrene crystal obtained by *ab initio* DFT calculation, the 9-mode model is generated by dropping the modes with $\lambda_m = g_{m,\mathrm{I}}^2 \omega_m < 20\ \mathrm{cm}^{-1}$ and adding λ_m of the dropped modes to the closest retained modes, while the 4-mode model is generated in the same way except that the dropping threshold is upraised to 50 cm^{-1}[7]. Thus, the 9-mode model and the 4-mode model share the same total reorganization energy $\sum_m \lambda_m$ and the 9-mode model in principle describes realistic material better than the 4 mode-model.

In Fig. 5 we list the MPS parameters for which we have used the 9-mode model as well as

TABLE I. Vibration energy and local EPC constant of the 9-intramolecular-mode model.

Mode No.	1	2	3	4	5	6	7	8	9
ω_m (meV)	10	27	78	124	149	167	169	190	198
$g_{m,\mathrm{I}}$	0.96	0.38	0.25	0.20	0.15	0.31	0.13	0.20	0.31

the corresponding correlation functions $C(t)$. In Fig. 5(a) and (c) it can be seen that that the artificial recurrence around 6000 a.u. for the 4-mode model is absent in the 9-mode model. In addition, $C(t)$ of the 4-mode model and the 9-mode model agree well in the small t regime. For the $V = 5$ meV, $\Delta V = 10$ meV case and the $V = 20$ meV, $\Delta V = 0$ meV case shown in Fig. 5(b) and (d), $C(t)$ for the 4-mode model takes a long time to decay to zero, while $C(t)$ for the 9-mode model decays to zero in a faster manner.

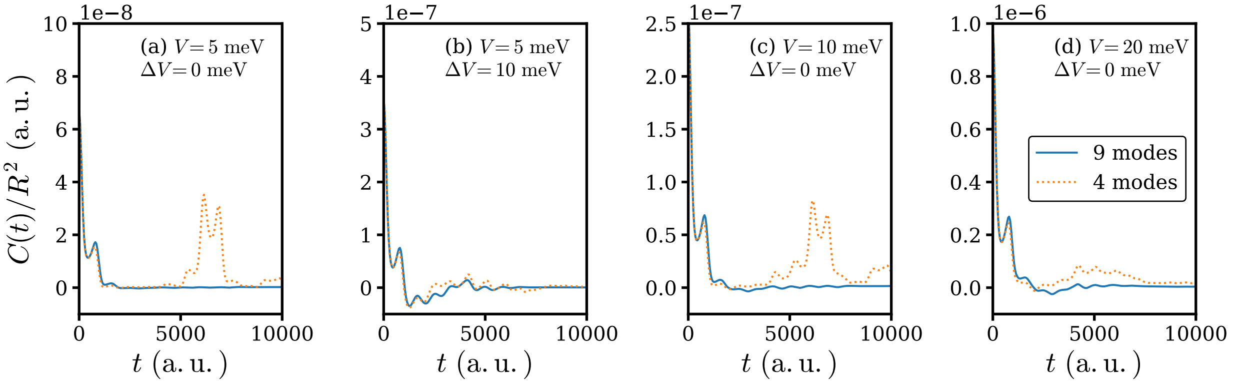

FIG. 5. Comparison of the current-current correlation function $C(t)$ obtained from the 4-mode model and the 9-mode model.

REFERENCES

[1] Borrelli, R. and Gelin, M. F. Quantum electron-vibrational dynamics at finite temperature: Thermo field dynamics approach, *J. Chem. Phys.* **145**, 224101 (2016).

[2] Tamascelli, D., Smirne, A., Lim, J., Huelga, S. F., and Plenio, M. B. Efficient simulation of finite-temperature open quantum systems, *Phys. Rev. Lett.* **123**, 090402 (2019).

[3] Fetherolf, J. H., Golež, D., and Berkelbach, T. C. A unification of the Holstein polaron and dynamic disorder pictures of charge transport in organic crystals, *Phys. Rev. X* **10**, 021062 (2020).

[4] Bussolotti, F., Yang, J., Yamaguchi, T., Yonezawa, K., Sato, K., Matsunami, M., Tanaka, K.,

Nakayama, Y., Ishii, H., Ueno, N., and Kera, S. Hole-phonon coupling effect on the band dispersion of organic molecular semiconductors, *Nat. Commun.* **8**, 173 (2017).

[5]Hannewald, K. and Bobbert, P. Ab initio theory of charge-carrier conduction in ultrapure organic crystals, *Appl. Phys. Lett.* **85**, 1535–1537 (2004).

[6]Troisi, A. Prediction of the absolute charge mobility of molecular semiconductors: The case of rubrene, *Adv. Mat.* **19**, 2000–2004 (2007).

[7]Li, W., Ren, J., and Shuai, Z. Finite-temperature TD-DMRG for the carrier mobility of organic semiconductors, *J. Phys. Chem. Lett.* **11**, 4930–4936 (2020).
